# Author's reply

**Published:** 2010

**Authors:** Monica Saini, Dheraj Khurana

**Affiliations:** Department of Neurology, PGIMER, Chandigarh, India; 1 Alchemist Hospital, Panchkula, India

Sir,

We thank Luis I. Gonzalez-Granado for his comments[1] regarding our case report.[2] Chronic relapsing optic neuropathy (CRION) is a recently described disorder, and there is a paucity of systematic studies evaluating the type/duration of immunosuppressive therapy required to maintain remission and preserve vision. Long-term steroids certainly have well-known adverse effects. Deflazacort is reported to cause less metabolic dysfunction in comparison to the usual oral steroids and is a safer option in patients requiring long-term steroids.[3] The adverse effects of oral steroids are known to be dose related.[4] In our patient, we were successfully able to taper steroids to a very low dose (1 mg of deflazacort on alternate days), following which steroids have presently been discontinued. Patients of CRION may, however, require long-term moderate to high doses of steroids in order to mantain remission. CRION is diagnosed once demyelinating disorders and autoimmune diseases have been excluded. Its steroid resposiveness argues in favor of a localized immune pathophysiology. Corticosteroid-sparing immunosupressive agents and intravenous immunoglobulins (IvIg) have been reported to allow decrease in dose, and in some cases, discontinuation of oral steroids, in patients with corticosteroid-dependent autoimmune optic neuropathy and optic neuritis not associated with demyelinating disease.[5,6] Based on these observations, we agree that in patients of CRION, steroid-sparing agents including IvIg should be considered in those becoming steroid dependent.
